# Comparison of the Chemical Profiles and Antioxidant Activities of Different Parts of Cultivated *Cistanche deserticola* Using Ultra Performance Liquid Chromatography-Quadrupole Time-of-Flight Mass Spectrometry and a 1,1-Diphenyl-2-picrylhydrazyl-Based Assay

**DOI:** 10.3390/molecules22112011

**Published:** 2017-11-20

**Authors:** Xiaoming Wang, Jinfang Wang, Huanyu Guan, Rong Xu, Xiaomei Luo, Meifeng Su, Xiaoyan Chang, Wenting Tan, Jun Chen, Yue Shi

**Affiliations:** 1 Institute of Medicinal Plant Development, Chinese Academy of Medical Science & Peking Union Medical College, Beijing 100193, China; lmlwxm123@163.com (X.W.); jfwang105@163.com (J.W.); guanhuanyu630@163.com (H.G.); rxu@implad.ac.cn (R.X.); Luoxiaomei1019@163.com (X.L.); changxiaoyan1234@163.com (X.C.); jchen@implad.ac.cn (J.C.); 2 Beijing University of Chinese Medicine, Beijing 100102, China; 20160931861@bucm.edu.cn (M.S.); 18811790612@163.com (W.T.)

**Keywords:** cultivated *Cistanche deserticola*, phenylethanoid glycosides, antioxidant, UPLC-QTOF/MS

## Abstract

In this study, a sensitive ultra-performance liquid chromatography-photodiode array coupled to quadruple time-of-flight mass (UPLC-PDA-Q/TOF-MS) method and a 1,1-diphenyl-2-picrylhydrazyl (DPPH)-based assay were used to determine the chemical constituents and screen the antioxidant activity profiles of the methanol extracts of different parts of cultivated *Cistanche deserticola* (*C. deserticola*). First, qualitative and quantitative chemical composition analyses of the different parts of cultivated *C. deserticola* were conducted. Obvious differences were observed between the chemical profiles and content distribution of phenylethanoid glycosides (PhGs) from the different cultivated *C. deserticola* parts. The average contents of the six PhGs parts varied from 4.91 to 72.56 mg/g DW (milligrams of extract per gram of plant dry weight) in the six different parts of *Cistanche deserticola*, displaying a significant decreasing trend from the bottom to the top of cultivated *C. deserticola* and the highest content in the stems. From the bottom to the top of the plant, the echinacoside and cistanoside A content decreased and the 2′-acetylacteoside content increased. Second, an offline DPPH assay revealed that the total scavenging activities of all parts within the range of 20–500 µg/mL increased in a concentration-dependent manner and that good antioxidant activities were found in all plant parts, particularly in the stems, which could be related to their higher PhG content. Additionally, a DPPH-UPLC-PDA method was successfully applied to rapidly screen the antioxidant profiles and antioxidant components of the different cultivated *C. deserticola* parts. According to the antioxidant profiles before and after the DPPH reaction, there were wide variations in the antioxidant activities of different cultivated *C. deserticola* parts. Moreover, the antioxidant profiles revealed the presence of major free radical scavengers identified as PhGs using UPLC-Q/TOF-MS. Finally, the established DPPH-UPLC-PDA method was reagent saving, rapid and feasible for correlating the chemical profile of traditional chinese medicines (TCMs) with their bioactivities without isolation and purification and may be used for multicomponent analysis of active substances in other foods and herbs. Therefore, to better harness *C. deserticola* resources, using this method to evaluate cultivated *C. deserticola*, a promising herb material with obvious antioxidant activity, is crucial.

## 1. Introduction

**Figure 1:**
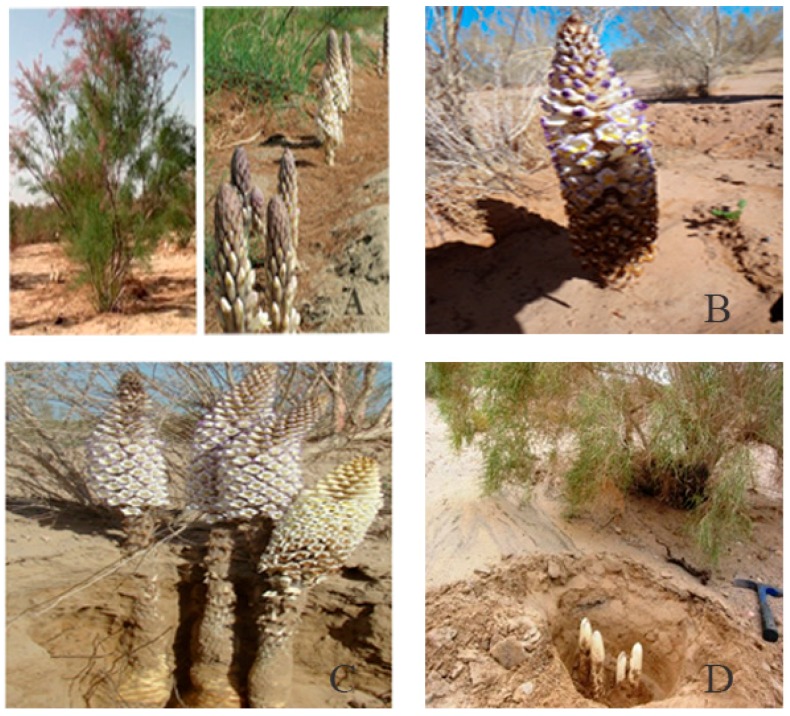
The growth environment (**A**); flowers- the aerial parts (**B**); the whole plants (**C**) and succulent stem collections (**D**) of *Cistanche deserticola*.

*Cistanche deserticola* (called “Rou Cong Rong” in Chinese, CH), one of the most precious traditional Chinese medicines (TCMs) recorded in Shen Nong’s Materia Medica (A.D. 102–200), nourishes the kidneys, strengthens the “yang” and has remarkable curative effects on kidney deficiency, female infertility and senile constipation [1]. *C. deserticola*, a holoparasitic plant, is found in the desert region of northwestern China in addition to similar regions of countries such as Iran, India, and Mongolia (Figure 1). The dried fleshy stem of *C. deserticola* has been commonly used as a tonic in China and Japan for many years to treat kidney deficiency, impotence, female infertility, profuse metrorrhagia and senile constipation [2]. Modern pharmacological studies have demonstrated that its extracts can enhance a person’s learning and memorization ability, treat Alzheimer’s disease, improve immunity, exert anti-ageing and anti-fatigue effects and promote bone formation [3]. *C. deserticola* has earned the honour of being called the “ginseng of the desert” in China because of its ginseng-like tonic effects and has been widely accepted as a health supplement in Japan, Korea and many other countries because of its remarkable and reliable medical benefits [4,5,6]. In Xinjiang, China, local people use *C. deserticola* stems to make tea for tonifying kidney and invigorating yang as a result of its nutritional and health effects. *C. deserticola* phytochemicals and their products are generally nontoxic and possess many medicinal benefits. Chinese medicines containing *C. deserticola* in different formulations have been developed and patented. Therefore, these plants are important crops for rural communities in desert regions.

To date, various phenylethanoid glycosides (PhGs) have been isolated from *C. deserticola* and its related species (*Cistanche tubulosa* and *Cistanche salsa*) [7]. PhGs, which are the major active constituents of this plant, were reported to have a strong antioxidative effect [8]. Hence, *C. deserticola* extracts may be beneficial to the food, agriculture, nutraceutical, cosmetics and pharmaceutical industries. However, the natural resource of *C. deserticola* is near extinction because of unrestricted digging; therefore, *C. deserticola* has been protected as a vulnerable plant in China. To meet increasing demand for *C. deserticola* from the international and domestic herbal medicine markets, *C. deserticola* has been cultivated in several plantations located in Inner Mongolia, Ningxia and Xinjiang. Further research and development of cultivated *C. deserticola* is of great significance to protect of wild *C. deserticola* resources, strengthen environmental management in the desert regions, and promote local economic development. However, research on cultivated *C. deserticola* is still lagging behind that on its active ingredients. In addition, few studies have focused on other parts of *C. deserticola* other than the succulent stem.

Several conventional high performance liquid chromatography (HPLC) methods have been successfully established for qualitative and quantitative analysis of *C. deserticola* [9,10,11,12]. Currently, little is known about the distribution of PhGs in the different parts of *C. deserticola*. Because ultra-performance liquid chromatography coupled to quadruple time-of-flight mass (UPLC-QTOF-MS) has excellent separation and good structural characterization abilities, this technique is suitable for analysing the complex extracts used in TCMs. Hence, some authors have successfully used UPLC/Q-TOF to analyse the chemical constituents of *C. deserticola* [13,14,15]. However, the chemical composition of different parts of *C. deserticola* has not been elucidated in detail. Studies on the chemical profiles of different parts of this plant are necessary for the comprehensive development and utilization of medicinal materials derived from this plant. In recent years, the 1,1-diphenyl-2-picrylhydrazyl (DPPH) radical scavenging assay has been employed to investigate the antioxidant activity of TCMs. Furthermore, a sensitive DPPH-HPLC method has been developed to screen the profiles related to antioxidant activity [16,17,18,19]. This method was successfully applied to screen and identify the natural bioactivity of complex mixtures and rapidly highlight peaks in chromatograms that correspond to bioactive compounds. Few previous studies [20,21] have found that the crude extracts of *C. deserticola* exhibit antioxidant properties, which might be related to their PhG constituents. However, none of these studies have comprehensively evaluated the combination of the chemical components and bioactivity of *C. deserticola*. To the best of our knowledge, there is also no prior report on the phytochemical composition or antioxidant activities of different parts of *C. deserticola*. Thus, comparing the phytochemical composition of the different parts of cultivated *C. deserticola*, linking these findings with antioxidant activity and combining the results with a correlation analysis is of great significance.

The objective of this study was to evaluate the chemical profiles and antioxidant activities of the different parts of cultivated *C. deserticola* using a DPPH-UPLC-PDA assay coupled with UPLC-QTOF-MS/MS. For the first time, the antioxidant profiles of six parts of cultivated *C. deserticola* before and after the DPPH free radical scavenging reaction were screened and compared. The major active compounds were identified as phenylethanoid glycosides, including echinacoside, cistanoside A, acteoside, 2′-acetylacteoside, tubuloside B and isoacteoside and their possible fragmentation pathways were proposed. The antioxidant contents of the six different parts of cultivated *C. deserticola* were also simultaneously determined using UPLC-PDA. The antioxidant activity and chemical composition profiles of the six parts of cultivated *C. deserticola* were studied as well.

## 2. Results and Discussion

### 2.1. Optimization of Extraction and UPLC Conditions

The conditions for sample pretreatment were optimized by investigating different concentrations of methanol solution (50%, 70%, 100%, *v*/*v*), and extraction time (20, 30 and 60 min). As a result, 100% methanol was found to be an ideal extraction solvent because it extracted the most marker components in relatively high yields. Studying the influence of the extraction time on the extraction efficiency showed that most marker compounds were extracted in the highest yields within 30 min. Ultrasonication times longer than 30 min did not substantially increase the quantity of the components in the extracts. Several chromatographic parameters, including mobile phase composition, wavelength and column temperature, were optimized for the UPLC analysis to achieve high resolution as quickly as possible. Compared to methanol–water, acetonitrile–water afforded a higher resolution. Adding 0.2% (*v*/*v*) FA at a column temperature of 30 °C with a mobile phase flow rate of 0.4 mL/min provided satisfactory baseline peak shape and resolution. Adequate separation was achieved within 18 min by operating the UPLC under gradient elution conditions, as described in Section 3.4. Considering the number of peaks, quantification and sensitivity, the detector wavelength was set at 330 nm.

### 2.2. Analysis of UPLC Fingerprints and Similarities

Relative retention time, relative peak area and similarities were used to evaluate the quality of the fingerprints. The precision was determined via six consecutive injections of the same sample solution. The relative standard deviations (RSDs) of the relative retention time and relative peak area of the common peaks were all below 0.87% and 1.47%, respectively, and the similarities of different chromatograms were all above 0.95. The repeatability was evaluated by analysing six reduplicative samples. For these samples, the RSDs of relative retention time and relative peak area of the common peaks were all below 1.59% and 1.97%, respectively, and the similarities of different chromatograms were all above 0.95. Stability was assessed by testing one sample solution at different times (0, 2, 4, 6, 8, 12 and 24 h) over 24 h. The RSDs of the relative retention time and relative peak area of the common peaks were all below 0.96% and 1.98%, respectively, and the similarities of different chromatograms were all above 0.95. All these results indicated that the samples remained stable during the testing period and that the fingerprint analysis conditions were satisfactory.

**Figure 2:**
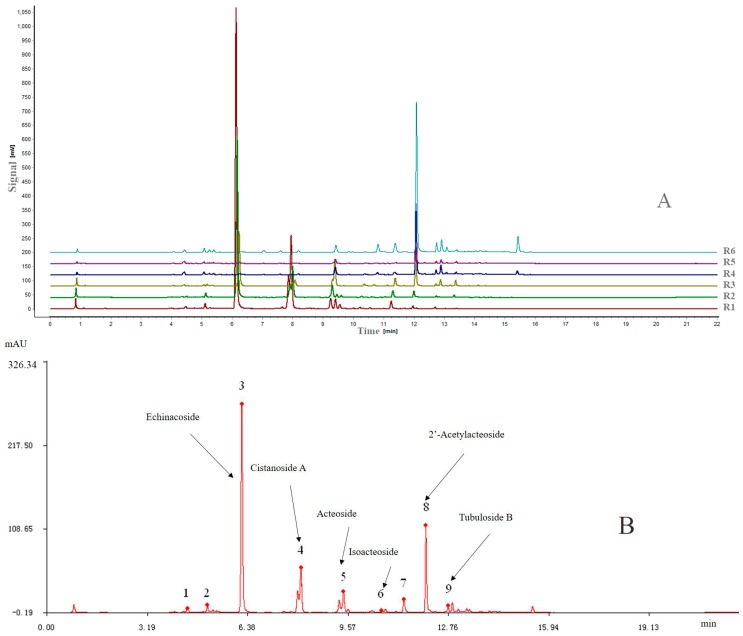
(**A**) Overlaid UPLC chromatograms of samples from Nos. R1–R6; (**B**) the reference chromatogram (marked R) was obtained by using SES for Chromatographic Fingerprint of TCMs.

Similarity analysis (SA) of the extracts of the six different parts of cultivated *C. deserticola* was conducted under optimal conditions and matched automatically using a Similarity Evaluation System (SES) for Chromatographic Fingerprint of Traditional Chinese Medicine software (Version 2004 A; Manufacturer, Beijing, China), which was recommended by the China Food and Drug Administration (CFDA). Then, a reference chromatogram (R) was generated by this software by comparing the six different extract chromatograms using the median method. The fingerprints revealed the presence of nine common peaks, which were assigned as “characteristic peaks”, in the chromatograms of the six extracts from cultivated *C. deserticola*. Figure 2A shows the overlay chromatograms of the six different part extracts at 330 nm, and Figure 2B shows the reference chromatogram of the sample with the nine marker compounds.

All samples differed in chromatographic patterns and peak abundances. Furthermore, there were still some differences between the chemical fingerprints of the different parts, including peak numbers and peak abundances. The similarities between the entire chromatographic profiles of the six different part extracts and the reference chromatogram were evaluated by SES software and their correlation coefficients of their chemical fingerprints are shown in Table 1. The results showed that the correlation coefficients of these samples ranged from 0.07 (R6) to 0.993 (R2), which indicated that obvious chemical composition differences existed between the different parts of cultivated *C. deserticola*. The differences in the correlation coefficients indicated variation in the fingerprint and internal qualities of these samples.

### 2.3. Quantitative Analysis of Six Phenylethanoid Glycosides (PhGs) by UPLC-PDA

**Figure 3:**
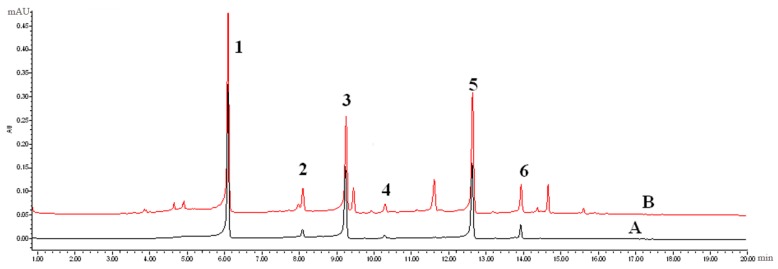
Typical UPLC-PDA chromatograms of mixed standards (**A**) and samples (**B**).

An UPLC-PDA method for the simultaneous determination of six PhGs in the different parts of cultivated *C. deserticola* was established. A 2 μL aliquot of the working solutions was injected into UPLC for analysis. The UPLC-PDA chromatograms of the mixed standards and samples under the optimized conditions described in Section 3.4 are shown in Figure 3. The proposed UPLC-PDA quantitative analysis method was validated by determining the linearity, limit of detection (LOD), limit of quantification (LOQ), intra- and inter-day precisions, repeatability, stability and accuracy.

The calibration curve for each compound was established by plotting the peak area (*y*) versus the concentration (*x*) of each analyte at seven concentrations run in triplicate (*n* = 3). The limit of detection (LOD) and the limit of quantitation (LOQ) for the six PhGs were determined at signal-to-noise ratios (S/N) of 3 and 10, respectively, by injecting a series of dilute solutions of known concentrations. All of the compounds showed good linearity (r^2^ > 0.9995) within the linear range. The calibration curves, linear range, r^2^ value, LOD and LOQ are listed in Table 2. The intra- and inter-day precisions were investigated by analysing a mixed PhG standard solution injected six times (*n* = 6) during a single day and by duplicating the experiment on three successive days. To further confirm the repeatability of the developed assay, six independent working solutions, all of which were prepared from Sample R1, were analysed using the above method. The stability was analysed by injecting Sample R1 at room temperature at 0, 6, 12, 24, 48 and 72 h. The RSD was calculated as a measurement of the precision, repeatability and stability. Accuracy was determined using the recovery percentage. The recovery test was determined by spiking a known amount of the six PhGs into a sample with a known concentration and then extracting and analysing the resulting solution using the same procedures. Six replicates were performed for this test. The ratio of the determined amount and the added amount was used to calculate the recovery. As shown in Table 3, the RSDs of the intra-and inter-day variations, repeatability and stability of the six PhGs were less than 2%, and the average recoveries were between 97.6% and 102.2% with an RSD of less than 3%. The abovementioned method validation results demonstrated that our method was precise, accurate and sensitive enough for simultaneous quantitative analysis of the six PhGs in different parts of cultivated *C. deserticola*.

**Figure 4:**
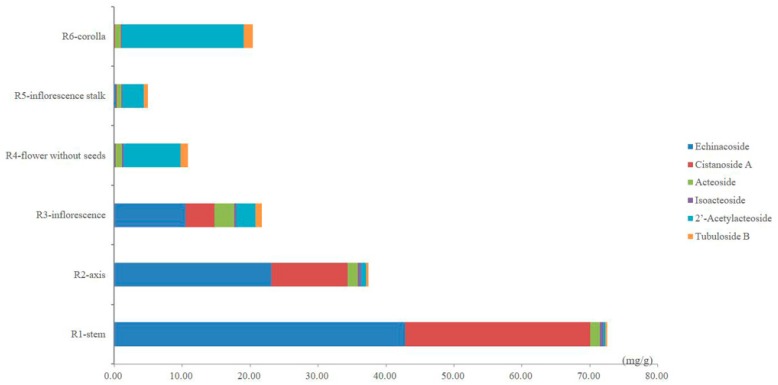
The bar graph of the average contents of six PhGs in samples from six different parts of cultivated *C. deserticola*.

The contents of the six PhGs in the different parts of cultivated *C. deserticola* are summarized in Table 4 as the average values of three replicate injections. As shown in Table 4, remarkable differences between the contents of the six PhGs in the different parts were observed, suggesting that each part has its own chemical characteristics. The total content of the six PhGs ranged considerably from 4.91 to 72.56 mg/g and decreased significantly from the bottom to the top of cultivated *C. deserticola*. Between the different parts, the total contents of the six PhGs approximately 3–20-fold. The highest value was found in the stems (72.56 ± 0.96 mg/g), whereas the lowest value was observed in the inflorescence stalks (4.91 ± 0.22 mg/g). In addition, the content of each determined PhG was found to differ strongly between these different parts. As shown in the comparison between the different parts in Figure 4, echinacoside (10.51–42.80 mg/g, the main compound), cistanoside A (4.21–27.28 mg/g) and acteoside (1.37–2.96 mg/g) were the three most abundant ingredients, whereas relative minor distributions were observed for isoacteoside (0.32–0.51 mg/g), 2′-acetylacteoside (0.37–2.83 mg/g) and tubuloside B (0.23–0.89 mg/g) in the stems, axes and inflorescences. However, in the flowers (without seeds), inflorescence stalks and corollas, 2′-acetylacteoside (3.16–17.89 mg/g, the main compound), tubuloside B (0.6–1.36 mg/g) and acteoside (0.67–0.93 mg/g) were found in relatively higher amounts, whereas echinacoside (0.09–0.31 mg/g), cistanoside A (0.03–0.07 mg/g) and isoacteoside (0.17–0.23 mg/g) were scarcer. Thus, our data are helpful for obtaining a better understanding of the differences in PhG distribution between these different parts. Interestingly, from the bottom to the top of cultivated *C. deserticola*, the echinacoside and cistanoside A content decreased significantly; by contrast, the 2′-acetylacteoside content increased. These results highlight the need for proper quality control of cultivated *C. deserticola*.

### 2.4. Antioxidant Capacity of Different Parts of Cultivated *C. deserticola* Evaluated by Off-Line DPPH Assay

**Figure 5:**
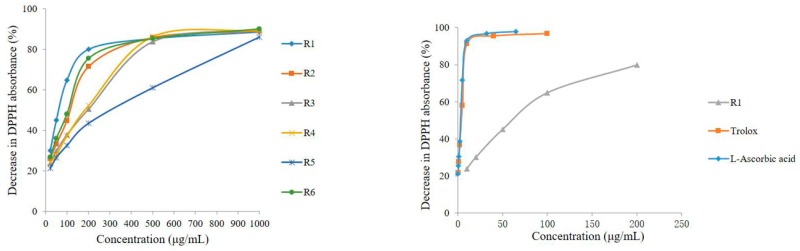
The DPPH free-radical scavenging capacity of six different parts of *C. deserticola* and the comparisons among sample R1 and reference antioxidants of Trolox and l-Ascorbic acid.

Previous studies [8] have shown that PhG aglycone is a powerful antioxidant. The free radical scavenging activity of DPPH is extensively used to evaluate the radical scavenging ability of natural antioxidants. In the present study, the total antioxidant activities of the different parts of *C. deserticola* were assayed using an offline DPPH method. The percentage decrease in DPPH absorbance of the samples at various concentrations is shown in Figure 5, which reveals that the abilities to scavenge the DPPH radical of the six different parts of cultivated *C. deserticola* as well as those of the standards (Trolox and l-ascorbic acid), behaved in a concentration-dependent manner and were varied. Accordingly, the IC_50_ values, i.e., the sample concentrations required to scavenge 50% of DPPH radicals, were determined to measure the total antioxidant capacity of the different parts of the cultivated plant. Lower IC_50_ values indicate stronger antioxidant activities. The results, as shown in Table 5, indicated that all extracts had good dose-dependent inhibitory activity against the DPPH radical, although all of them were less potent than the reference antioxidants Trolox and l-ascorbic acid (IC_50_ values of 2.18 ± 0.03 and 1.78 ± 0.01 mg/mL, respectively). Among the extracts, the R1 (stem) and R5 (inflorescence stalk) extracts showed the highest (IC_50_ of 53.659 ± 1.04) and lowest antioxidant activity (IC_50_ of 202.46 ± 2.98 μg/mL), respectively. The results also revealed that the order of antioxidant potency (IC_50_) of the six parts was, in decreasing order: stem > corolla ~axis > flower (without seeds) ~inflorescence > inflorescence stalk. According to previous reports [20,21], the stem extracts of *C. deserticola* possess good antioxidant activities. Furthermore, PhGs were claimed to be responsible for the antioxidant activity because of their hydroxyl groups. Similarly, our results reveal that there are abundant antioxidative phytochemicals in the extracts of cultivated *C. deserticola*, especially in the stem extract. In addition, our results indicated that there may be a positive relationship between composition, PhG content and antioxidant activity.

### 2.5. UPLC-PDA Coupled with Pre-Column DPPH Assay for Screening Antioxidant Profiles

The UPLC coupled with a pre-column DPPH assay has successfully combined the determination of active compounds with the free radical scavenging capability to evaluate the quality of herbal medicines, and could reflect the bioactivity of the herbal medicines directly [22,23]. Therefore, this method would be superior to multicomponent quantification for evaluating herbal medicine quality.

The extract with the best antioxidative activity (Sample 1, R1) was further screened using UPLC-PDA coupled with the pre-column DPPH assay. First, 1 mL of the R1 methanol solution (12 mg/mL) and 2 mL of 3 mM DPPH in methanol were mixed and reacted for 60 min. Both the reaction mixture and R1 methanol solution were then analysed using UPLC-PDA under the same chromatographic conditions. Subsequently, comparing to the chromatogram of the reaction mixture to that of R1 revealed that some peaks had disappeared or decreased in height and therefore could be considered as antioxidant peaks. Radical scavenging profiles revealed the six PhG peaks in chromatographic fingerprint possessing obvious free radical inhibition effects.

**Figure 6:**
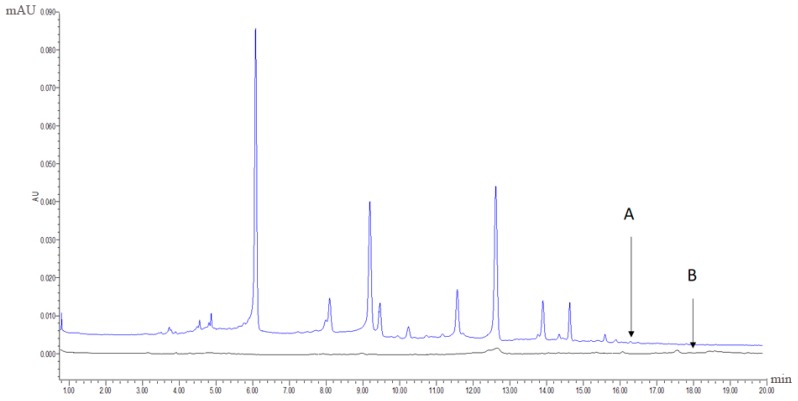
(A) the chromatographic fingerprint of Sample 1 (R1) and (B) the antioxidant activity fingerprint of Sample 1 (R1) after the DPPH free radical scavenging reaction detected at 330 nm, respectively.

Figure 6A,B display the chromatographic fingerprint of Sample 1 (R1) and the antioxidant activity fingerprint detected at 330 nm, respectively. The disappeared or reduced peaks in the activity fingerprint indicate that these components have free radical scavenging activity. Clearly, antioxidant activity was observed for the marker compounds found in the chromatographic fingerprint, indicating that the antioxidant activity of cultivated *C. deserticola* is attributable to the presence of PhGs. The other five parts showed similar antioxidant activity fingerprints (not shown). UPLC-PDA coupled with the pre-column DPPH assay has a clear advantage over the offline assay method because the individual contribution to the total antioxidant activity of each chemical component can be determined, and the main radical scavengers can be further identified by HPLC-MS.

### 2.6. Identification of Six Phenylethanoid Glycosides (PhGs) by UPLC-ESI-QTOF/MS

According to previous studies, the structures of the PhGs in cultivated *C. deserticola* have several similarities [7]. The empirical structural features of these PhGs are as follows: for the disaccharide glycosides, the sugar moiety comprises glucose (Glc) and rhamnose (Rha) connected by a Glc (3→1) Rha linkage, the Glc unit typically links directly to the aglycone, and a coumaroyl or caffeoyl moiety is usually located at the C4 or C6 position. For the trisaccharide glycosides, there is another Glc or Rha unit at the C6 position of the inner Glc unit. Under the optimized MS conditions, negative-ion mode was used to identify the six antioxidant peaks, which were further validated by comparing their retention time, UV/Vis data and MS data with those of standards. Peaks 3, 4, 5, 6, 8 and 9 detected in the fingerprint chromatograms were tentatively identified as echinacoside, cistanoside A, acteoside, isoacteoside, 2′-Acetylacteoside and tubuloside B, respectively, all of which are PhGs. Their retention time (RT) and MS and MS/MS data are illustrated in Table 6.

As shown in Table 6, peak 5 and peak 6 (retention times 10.99 and 12.35 min), which have the same predominant deprotonated ion at *m*/*z* 623 and fragment ions at *m*/*z* 461, 315, 179, 161 and 135, were identified as acteoside and isoacteoside by comparing their retention times and mass spectra with those of authentic standards. Both peaks 8 and 9 (retention times 14.44 and 15.09 min) exhibited an ion at *m*/*z* 665, which was 42 Da (the molecular weight of the acetyl (Ac) group) higher than those of peaks 5 and 6, and displayed a fragment ion at *m*/*z* 623 in the MS/MS spectrum corresponding to the loss of the Ac moiety (42 Da). Similar characteristic fragment ions, such as *m*/*z* 461, 315, 179, 161 and 135, were also obtained in the MS/MS spectra of the two peaks; therefore, we finally established peaks 8 and 9 to be 2′-acetylacteoside and tubuloside B, respectively, as they share the same retention times and mass spectra with the authentic reference standards. As peaks 5, 6 and peaks 8, 9 were only substituted positional isomers, the fragment ions were almost the same, preventing the MS from distinguishing between them. Fortunately, peaks 5, 6 and peaks 8, 9 were isolated as reference compounds and could be identified by their distinct UPLC retention times. The ion of peak 3 (retention time 7.22 min) was 162 Da (molecular weight of Glc) higher than those of peaks 5 and 6. The peak also showed characteristic fragment ions at *m*/*z* 623 in addition to at *m*/*z* 461, 315, 179, 161 and 135, and the fragment ion at *m*/*z* 623 was attributed to the loss of a Glc unit from the predominant deprotonated ion at *m*/*z* 785. It was identical to that of echinacoside, and its identity confirmed by comparing the retention time and mass spectra with those of the authentic reference standard. Peak 4 (retention time 8.88 min) had a predominant deprotonated ion at *m*/*z* 799, which was 14 Da (CH2) higher than that of peak 3, and fragment ions at *m*/*z* 637 and 475 (14 Da higher compared with the fragment ions at *m*/*z* 623 and 461). Additionally, the lower molecular weight characteristic fragment ions (lower than 200 Da), including *m*/*z* 179, 161 and 135, were the same as those mentioned above. It was determined as cistanoside A, which corresponds to the retention time and mass spectra of the authentic reference standard. Thus, the peak was determined as cistanoside A, as its retention time and mass spectra correspond to those of the authentic reference standard. Finally, the antioxidants were identified by their mass spectra and fragmentation patterns with the aid of standard references and literature reports [24,25,26,27,28,29,30,31,32].

**Figure 7:**
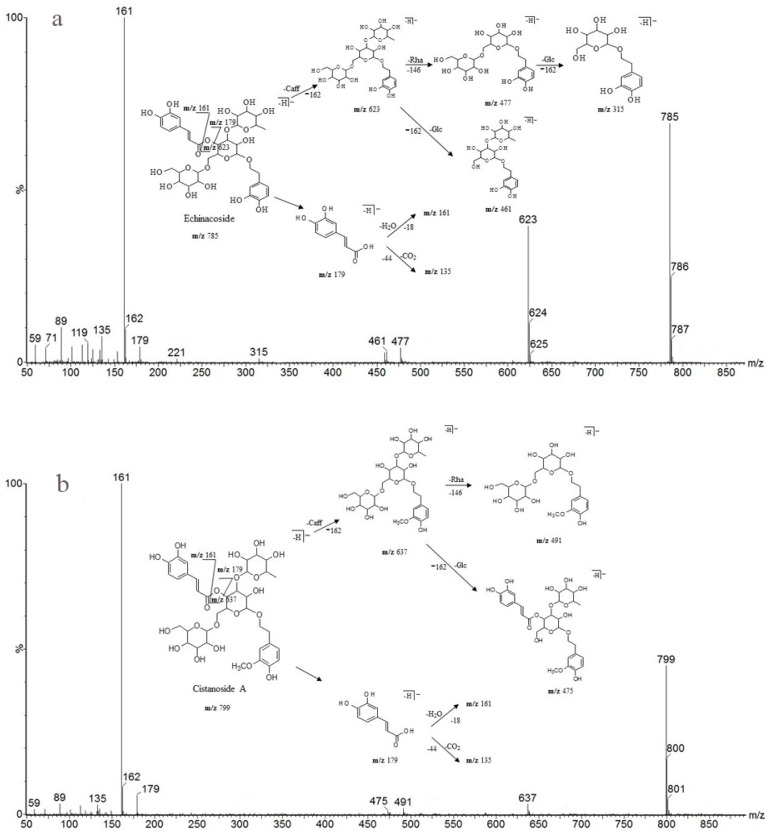

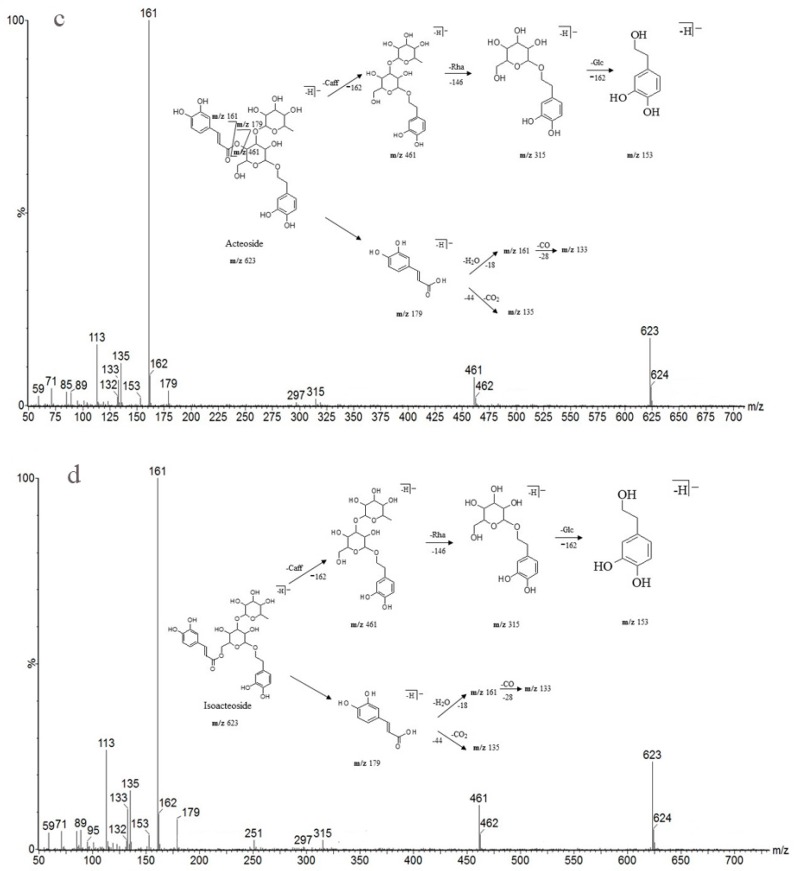

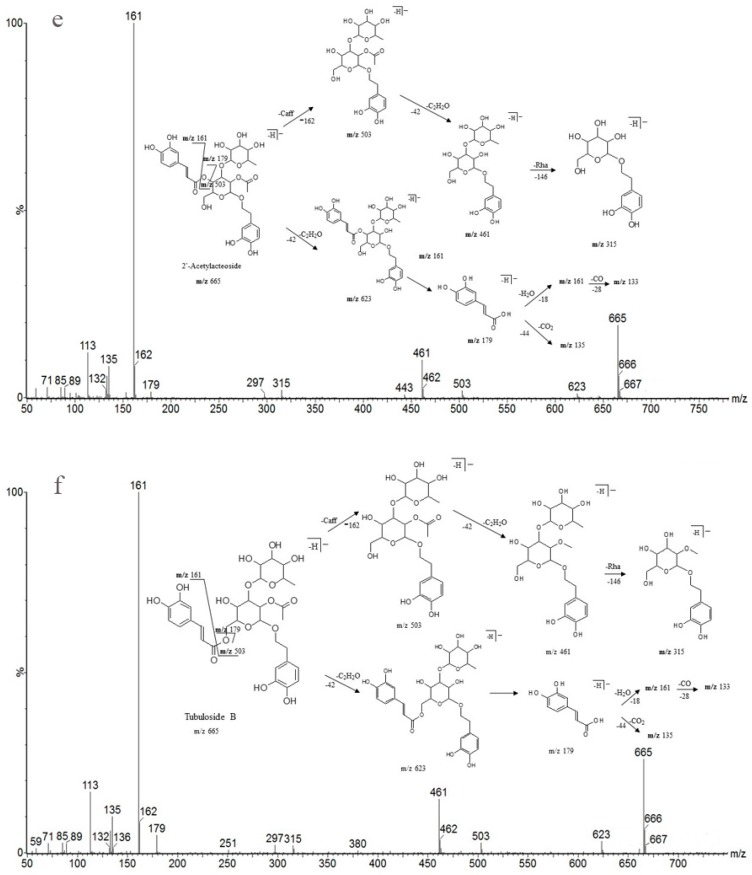
Detailed MS spectra and the proposed fragmentation patterns of Echinacoside (**a**); Cistanoside A (**b**); Acteoside (**c**); Isoacteoside (**d**); 2′-Acetylacteoside (**e**); Tubuloside B (**f**).

**Figure 8:**
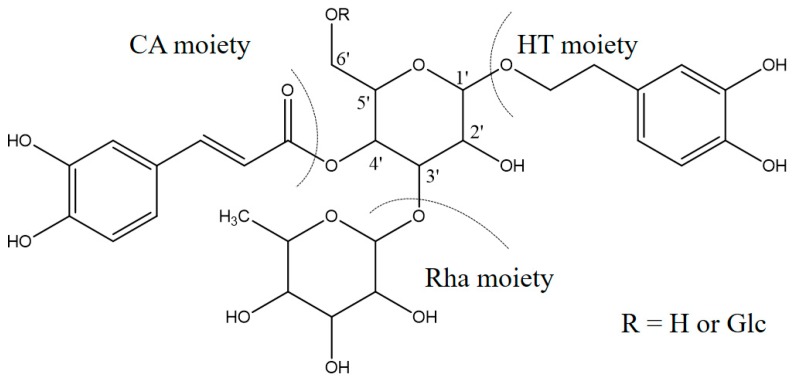
The common fragmentation positions of these PhGs.

Detailed mass spectral data and the proposed fragmentation patterns were given in Figure 7. The structures of the six PhGs identified above are divided into three moieties: I-caffeic acid (CA), II-aglycone and III-sugar (Glc and Rha). In the MS/MS spectra, the mass defects corresponding to the neutral cleavages of a caffeoyl group and Glc and Rha residues dominate the fragmentation pathways of PhGs and generate the diagnostic fragment ions (Figure 8). Additional MS/MS characteristic fragment ions at *m*/*z* 179, 161 and 135 further suggested that a caffeoyl substituent exists in their structures. Using these fragmentation features, we could confidently propose fragmentation pathways as follows. Acteoside (Figure 7c) produced a fragment at *m*/*z* 461 via the loss of a CA moiety and then produced an ion at *m*/*z* 315 via a further loss of Rha. The CA moiety was found at *m*/*z* 179, and its fragment ions found at *m*/*z* 161 and *m*/*z* 135 were produced by the loss of H_2_O and CO_2_, respectively. Isoacteoside (Figure 7d) produced the same ions as acteoside at *m*/*z* 461, 315, 179, 161, and 135. 2′-Acetylacteoside (Figure 7e) produced a fragment at *m*/*z* 623 via the loss of acetyl and then generated ions at *m*/*z* 461, 315, 179, 161 and 135, which was identical to the fragmentation pattern of acteoside. Meanwhile, 2′-acetylacteoside also produced a fragment at *m*/*z* 503 by losing its CA moiety and subsequently lost its Ac group to produce the ion at *m*/*z* 461. Tubuloside B (Figure 6f) displayed a similar fragmentation pattern to 2′-acetylacteoside. Echinacoside (Figure 7a) produced a fragment ion at *m*/*z* 623 via loss of its CA moiety, and then sequential losses of Rha and Glc moieties yielded the fragment ions at *m*/*z* 477 and 315. The fragment ion at *m*/*z* 461 was generated by losing the CA and Glc moieties from the precursor ion. The characteristic CA ions at *m*/*z* 179, 161 and 135 were also found. Cistanoside A (Figure 7b) produced a fragment ion at *m*/*z* 637 via loss of its CA moiety and then generated ions at *m*/*z* 491 and 475 by the further loss of Rha and Glc moieties, respectively. Therefore, the use of UPLC-PDA-QTOF/MS in combination could make a major contribution towards precisely elucidating the chemical structures of compounds.

### 2.7. Correlation between Antioxidants and A8ntioxidant Properties

**Figure 9:**
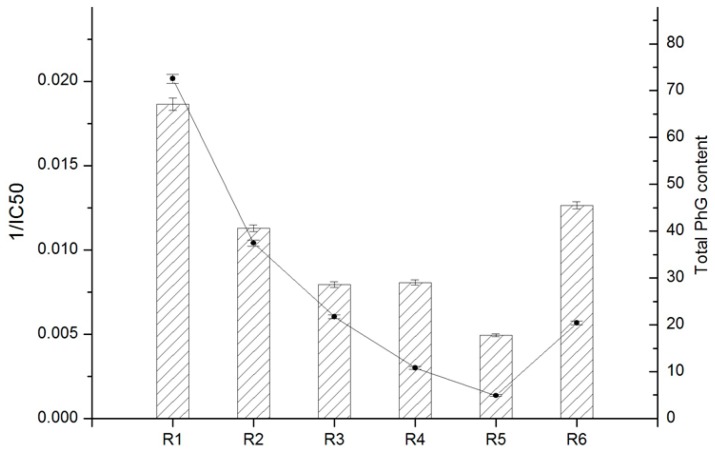
Comparison of the total PhG content and DPPH radical scavenging activity (1/IC_50_) of six different parts from cultivated *C. deserticola*. Data are presented as mean ± SD of each of three replicates (*n* = 3).

Correlation analysis between the total contents of antioxidants and total antioxidant activities has been described in many studies [33,34,35,36,37,38]. To the best of our knowledge, there is a lack of studies on comparing the antioxidant properties and PhG contents of cultivated *C. deserticola* parts using such a concise characterization method. The preliminary results of the six PhG contents described in the “Quantitative analysis” section are summarized in Table 4, and antioxidant activity (IC_50_) determined using the DPPH assay as described in the “Antioxidant capacity” section are listed in Table 5. First, the total antioxidant contents and activities of each parts are presented in Figure 9. Clearly, there are conspicuous differences between the different parts. In fact, the obtained results indicated that the antioxidant activity of cultivated *C. deserticola* stems was comparable with that of the stems available on the market. These results suggested that the antioxidant activity of the stem extract may be correlated to its PhG content. Therefore, the phytochemicals in the stem extract of cultivated *C. deserticola* are proposed to play an important role in its DPPH radical scavenging activity. Overall, our results indicated a strong correlation between total PhG content and antioxidant activity: r = 0.92, ** *p* < 0.01.

**Figure 10:**
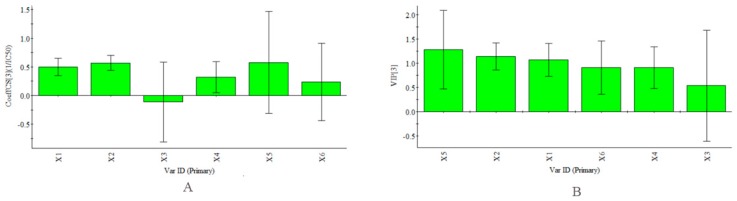
PLS Model (R2X = 0.990, R2Y = 0.998, Q2 = 0.982). (**A**) coefficients plot. The bars indicate 95% confidence intervals based on jackknifing; (**B**) VIP plot. Plots given by the SIMCA-P+ software (Version 13.0).

In addition, certain samples with higher total antioxidant contents did not show high antioxidant activities, such as the axis and inflorescence. Meanwhile, the corolla, which had the highest activity of the six parts, had a lower total antioxidant content than the axis and inflorescence. Figure 4, as described in the “Quantitative analysis” section, shows the relative amounts of the PhG compounds to give a preliminary understanding of their individual contributions. In this study, Partial Least Squares (PLS) regression analysis [39,40,41] was also carried out to compare the correlation between the abundance of each antioxidant and the antioxidant activity in vitro. The PLS model was constructed using the contents of each antioxidant (as the descriptor matrix X) in the fingerprint chromatograms at 330 nm and the antioxidant activities (as the response matrix Y) of all the parts. The coefficient plots and variable importance in projection (VIP) values obtained using SIMCA-P+ software (Version 13.0, Umetrics, Umea, Sweden) are shown in Figure 10. Notably, the inverse of the IC_50_ values (1/IC_50_) was selected as the Y variable to establish the models because lower IC_50_ values represent stronger antioxidant activity. According to the obtained regression coefficient plot in Figure 10A, the linear regression models show that all the antioxidants except acteoside were positively correlated with 1/IC_50_. The obtained calibration model of PLS is expressed by the regression equation: Y = 0.50X1 + 0.57X2 − 0.11X3 + 0.32X4 + 0.58X5 + 0.24X6. VIP values reflect the importance of variables in the model: the larger the VIP, the more relevant for sample classification. As our results showed, 2′-acetylacteoside was an important contributor to the antioxidant activity (Figure 10B). This finding was in agreement with a study by Yang et al [20].

## 3. Materials and Methods

### 3.1. Plant Materials

**Figure 11:**
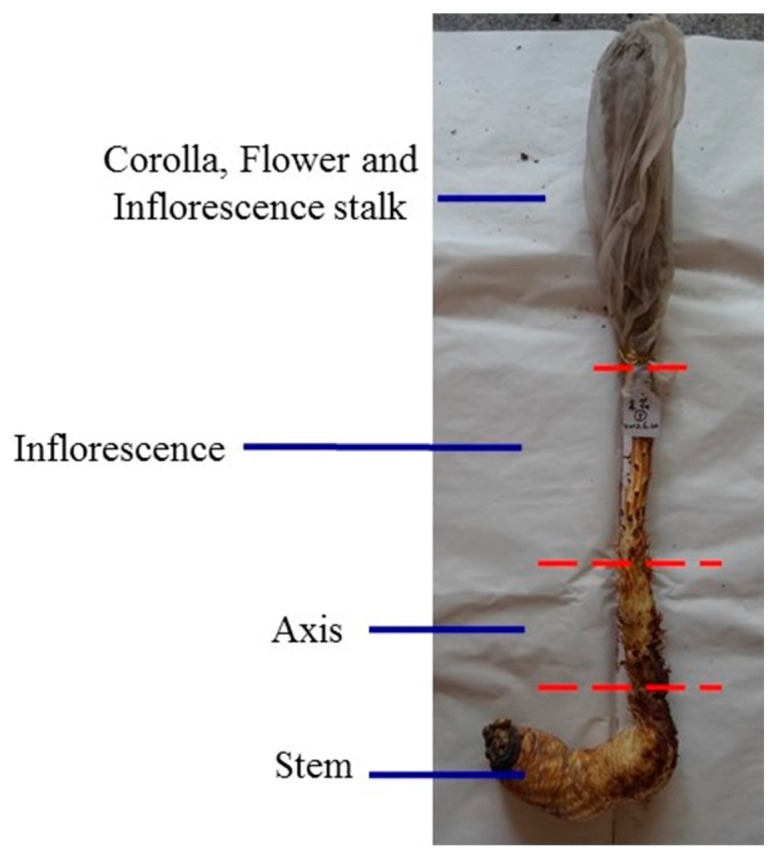

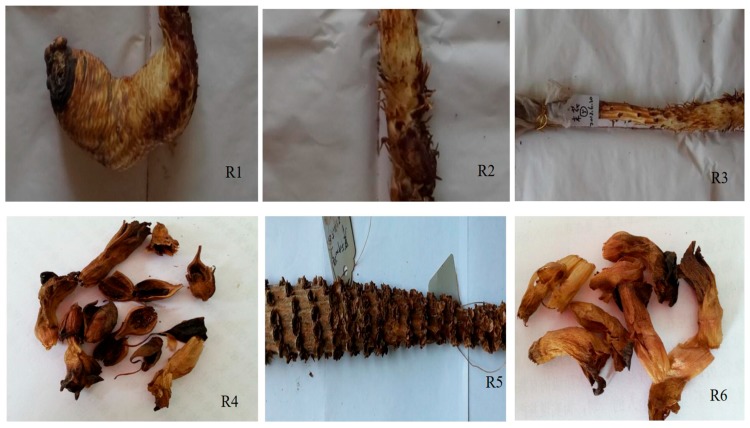
The six different parts of cultivated *C. deserticola* includes succulent stem (**R1**); inflorescence axis (**R2**); inflorescence (**R3**); flower (without seed) (**R4**); inflorescence stalk (**R5**) and corolla (**R6**).

Cultivated *C. deserticola* was generously provided by the Bencao Congrong planting base in Yongning County, Ningxia Province, and was authenticated by Prof. Jun Chen of the Institute of Medicinal Plant Development, Chinese Academy of Medical Sciences, Beijing, China. The six different parts of cultivated *C. deserticola* include the succulent stem (R1), inflorescence axis (R2), inflorescence (R3), flower (without seed) (R4), inflorescence stalk (R5) and corolla (R6). The detailed sample information is shown in Figure 11. Each part was dried separately in a dryer, crushed into powder, passed through a 40-mesh sieve, and then loaded into airtight containers and stored in a desiccator. Voucher specimens of these samples were deposited at Institute of Medicinal Plant Development, Chinese Academy of Medical Sciences, Beijing, China.

### 3.2. Chemicals and Reagents

Isoacteoside (Lot 130809), acteoside (Lot 130421) and echinacoside (Lot 121027) were purchased from Chengdu Pure-Chem Standard Co. Ltd. (Beijing, China), Cistanoside A, 2′-Acetylacteoside and tubuloside B were isolated and purified from cultivated *C. deserticola* in our laboratory, with more than 98% purity (HPLC) and their structure were also confirmed based on HR-MS, ^1^H-NMR and ^13^C-NMR and compared with previous literature. Their structures are displayed in Table 7.

DPPH, L-ascorbic acid and Trolox were purchased from Sigma-Aldrich (St. Louis, MO, USA). The solvents, acetonitrile and methanol, were of HPLC grade from Merck (Darmstadt, Germany), and formic acid with a purity of 96% was of HPLC grade (Tedia, Fairfield, OH, USA). Deionized water (18 MΩ) was prepared by distilled water through a Milli-Q system (Millipore, Milford, MA, USA). Other reagents and chemicals were of analytical grade.

### 3.3. Preparation of Solutions

#### 3.3.1. Standard Solutions

A mixed standard stock solution containing echinacoside (501 μg/mL), cistanoside A (64 μg/mL), acteoside (239 μg/mL), isoacteoside (22.6 μg/mL), 2′-acetylacteoside (278 μg/mL) and tubuloside B (46.2 μg/mL) was prepared in methanol. Stock solutions were diluted with methanol to obtain a series of appropriate concentrations that were used as working solutions to develop a calibration curve and were stored at 4 °C prior to use.

#### 3.3.2. Sample Solutions

An accurately weighed powder (40 mesh, 1 g) of six different parts of cultivated *C. deserticola* was added with 50 mL methanol, respectively. After being immersed for 30 min at room temperature, the mixtures were weighed and then extracted in an ultrasonic water bath (40 kHz, 250 W, Kunshan, China) at room temperature for 40 min. After cooling, additional methanol was added to compensate for the lost weight. All solutions were filtered through 0.22 µm membrane filters prior to injection. An aliquot (3 μL) of the supernatant solution was injected into the UPLC for analysis. Each extract was injected in triplicate.

#### 3.3.3. DPPH Solutions

The DPPH radical stock solution (5 × 10^−3^ mol/L) was prepared by dissolving an accurately weighed DPPH sample in methanol immediately before the experiments and was protected from light. Then, the stock solution was freshly diluted with methanol to obtain standard solution (1 × 10^−4^ mol/L).

### 3.4. UPLC-PDA Analysis Condition

UPLC analysis was conducted on a Waters Acquity UPLC system (Waters, Milford, MA, USA) comprising a column heater, a sample manager, a binary solvent manager and a photodiode array detector. Chromatographic separation was performed on an Acquity UPLC BEH C_18_ column (1.7 μm, 2.1 mm × 100 mm; Waters) by fixing the column heater at 30 °C. The mobile phase consisted of acetonitrile (A) and water containing 0.2% formic acid (B) at a flow rate of 0.4 mL/min. A gradient elution programme was employed as follows: 5% A at 0–2 min, 5–15% A at 2–4 min, 15% A at 4–6 min, 15–20% A at 6–10 min, 20–35% A at 10–15 min, 35% A at 15–18 min, and 35–5% A at 18–18.1 min. The composition was then held at 5% A for an additional 10 min for re-equilibration. The detection wavelength was set at 330 nm.

### 3.5. UPLC-ESI-Q/TOF-MS Analysis Condition

The UPLC conditions were the same as those described in Section 3.4. MS analysis was performed on a Waters Xevo G2-XS QTOF mass spectrometer (Waters MS Technologies, Manchester, UK) equipped with an electrospray interface (ESI) in negative-ion mode with a full scan MS spectrum over the *m*/*z* range of 50–1200. In the source, the capillary voltage was 2000 V, the source temperature was 120 °C, the desolvation temperature was 450 °C, the cone voltage was 40 V, the cone gas was 30 L/h and the desolvation gas flow rate was 600 L/h. MS/MS experiments were conducted using MS^E^, and the collision energy was 20–40 V. All MS data were collected using the Lock Spray system to ensure mass accuracy and reproducibility. The [M − H]^−^ ion of leucine-enkephalin at *m*/*z* 554.2615 was used as the lock mass in negative ESI mode. Data were acquired, analysed and processed using Waters Mass Lynx 4.1 software (Waters MS Technologies, Manchester, UK).

### 3.6. Offline DPPH Radical Scavenging Assay

The total antioxidant activity of the plant extracts was assessed using an offline DPPH radical scavenging assay. Sample solutions (2 mL) at various concentrations were mixed with 2 mL of 1 × 10^−4^ mol/L DPPH solution (in methanol). All samples were shaken and allowed to stand in the dark at room temperature for 60 min. The reduction in DPPH free radicals was measured by reading the absorbance at 517 nm against a blank (methanol without sample) on an ELISA reader (TECAN, Groding, Austria). Methanol (2 mL) alone was used as the control of this experiment. Trolox and l-ascorbic acid were used as the positive controls. The radical scavenging activity (% inhibition) of the tested samples, which was expressed as the DPPH scavenging percentage, was calculated using the following formula: % inhibition = [(Acontrol − Asample)/Acontrol] × 100. The total antioxidant activity was calculated by plotting the percent inhibition against the sample concentration and was represented as the sample concentration required to scavenge 50% of the DPPH radicals (IC_50_). All tests were carried out in triplicate, and the IC_50_ values were reported as the means ± SD.

### 3.7. UPLC-PDA Coupled with Pre-Column DPPH Assay

In this study, after 2 mL of DPPH solution (3 × 10^−3^ mol/L) and 1 mL of sample solutions (12 mg/mL) of different parts of cultivated *C. deserticola* were mixed, UPLC-PDA was employed to further screen the antioxidant profiles and identify the antioxidant components by detecting changes in the characteristic peaks of the sample solutions before and after the DPPH free radical scavenging reaction. For each different plant part, 10 µL aliquots of the sample solution before and after the DPPH assay were separately injected into the UPLC system under the same conditions as those described in Section 3.4. After the sample solutions were mixed with DPPH solution, changes in the characteristic peaks of the sample solutions were detected at 330 nm.

### 3.8. Data Analysis

Analytical data are expressed as the mean ± standard deviation (SD) of triplicate independent measurements. Bivariate correlation analysis was performed to study the relationship between the total PhG content and the antioxidant activity by determining the Pearson correlation coefficient (r) using IBM SPPS Statistics (Version 19; International Business Machines Corp., New York, NY, USA). The IC_50_ values were also calculated by probit analyses using SPPS Statistics 19. The DPPH free radical scavenging rate and total PhG content were plotted using OriginPro 8.5.1 (Origin Lab Corp., Northampton, MA, USA). Partial least squares (PLS) regressions were determined using SIMCA-P+ software (Version 13.0, Umetrics, Umea, Sweden).

## 4. Conclusions

Different parts of cultivated *C. deserticola*, a widely used herbal medicine, were investigated for their qualities. First, characteristic fingerprints of six different parts of the cultivated *C. deserticola* samples were generated and evaluated using SES and simultaneous analysis of the contents of six marker compounds. Second, quantifying the six major compounds using UPLC demonstrated the distribution characteristics of these compounds in the different parts. However, the results could not be directly related to the activity of the herbal medicine. Therefore, a better method for simultaneously determining the quality and activity of cultivated *C. deserticola* samples was necessary. First, we used an offline DPPH radical scavenging assay to evaluate the antioxidant activities of cultivated *C. deserticola* extracts and found the highest activity in the stem extract. Furthermore, UPLC-PDA coupled with the pre-column DPPH assay was applied to screen and evaluate the antioxidants in the cultivated *C. deserticola* stem extracts. This method not only provided more chemical information but also afforded some antioxidant information that could be used to identify and assess herbal medicines. Second, the active components in the stem extracts were separated and identified using UPLC-PAD-QTOF/MS to confirm the number and position of substituent groups and to improve the accuracy of the structural analysis. Finally, a relationship between the chemical components and in vitro antioxidant activity was established and validated using the PLS model.

In conclusion, for the first time, we have compared the chemical composition profiles and antioxidant activities of different parts of cultivated *C. deserticola* samples. These findings were of special interest since the distribution of PhG compounds, PhG content and their contribution to the antioxidant of different parts of cultivated *C. deserticola* samples have not been reported until the present study. Furthermore, PLS analysis was firstly established to give a preliminary understanding of six PhG’s individual contribution to the antioxidant activity. This approach may give us some new insights into comprehensive quality assessment of cultivated *C. deserticola*. Finally, this work represents the first report of a strong correlation between the DPPH radical scavenging activity and total PhG content of the different parts from cultivated *C. deserticola* (r = 0.92). Among these different parts, the stems were found to be rich in PhGs and to have strong antioxidant activity. In particular, the discarded aerial parts of cultivated *C. deserticola* also contained some bioactive compounds and might be used as an alternative dietary supplement. We hope that the results will provide useful information for future utilization of cultivated *C. deserticola*. To the best of our knowledge, this is the first report on the rapid identification and quantification of natural antioxidants in different parts of cultivated *C. deserticola* using UPLC-PAD-QTOF/MS, the offline DPPH method and the UPLC-PAD-pre-column DPPH method. According to our results, the method developed here could provide a powerful and meaningful tool for comprehensive quality control of complex herbal medicines. In our country, under the guidance of the “One Belt, One Road” initiative, this traditional crop with health benefits could be used to design new products with worldwide applications.

## Figures and Tables

**Table 1:** The Similarity analysis results of six different parts extracts from cultivated *C. deserticola*.

No.	R1	R2	R3	R4	R5	R6	R
R1	1	0.996	0.806	0.065	0.236	0.021	0.987
R2	0.996	1	0.824	0.092	0.261	0.048	0.993
R3	0.806	0.824	1	0.539	0.689	0.476	0.857
R4	0.065	0.092	0.539	1	0.923	0.988	0.119
R5	0.236	0.261	0.689	0.923	1	0.865	0.301
R6	0.021	0.048	0.476	0.988	0.865	1	0.070
R	0.987	0.993	0.857	0.119	0.301	0.070	1

**Table 2:** Calibration curves, linear range, LOD and LOQ for the six PhGs by UPLC-PDA.

No.	Analyte	Calibration Cures	r^2^	Linear Range (μg·mL^−1^)	LOD (μg·mL^−1^)	LOQ (μg·mL^−1^)
1	Echinacoside	*y* = 11006*x* − 12134	0.9999	10.02–501	0.06	0.21
2	Cistanoside A	*y* = 4029.4*x* − 442.01	0.9999	2.56–512	0.12	0.41
3	Acteoside	*y* = 12642*x* − 10163	0.9998	4.78–239	0.08	0.26
4	Isoacteoside	*y* = 5144.6*x* − 74.31	0.9998	2.26–452	0.11	0.35
5	2′-Acetylacteoside	*y* = 11443*x* − 5032.8	0.9999	5.56–556	0.05	0.18
6	Tubuloside B	*y* = 10153*x* − 2349.8	0.9995	2.30–369.60	0.10	0.31

**Table 3:** Validation of method for identifying six PhGs by UPLC-PDA.

Analyte	Precision (RSD, %)		Repeatability	Stability	Recovery (%, *n* = 3)	
	Intraday (*n* = 6)	Interday (*n* = 6)	(RSD, %, *n* = 6)	(RSD, %, *n* = 6)	Mean	RSD (%)
Echinacoside	0.55	1.44	1.37	1.90	98.80	0.86
Cistanoside A	0.99	1.40	0.78	0.94	100.10	2.23
Acteoside	0.95	1.52	1.92	1.71	102.20	1.33
Isoacteoside	1.32	1.50	1.11	0.92	97.60	2.13
2′-Acetylacteoside	0.68	1.26	1.88	1.59	101.20	1.10
Tubuloside B	0.79	1.77	1.06	1.89	99.10	2.92

RSD = (SD/mean) × 100%.; Recovery = (amount found − original amount)/spiked amount × 100%.

**Table 4:** The average contents of the six PhGs in the six different parts of *Cistanche deserticola*.

Sample	Content (mg/g, *n* = 3)						Total
	Echinacoside	Cistanoside A	Acteoside	Isoacteoside	2′-Acetylacteoside	Tubuloside B	
R1	42.80 ± 1.15	27.28 ± 0.61	1.37 ± 0.07	0.51 ± 0.03	0.37 ± 0.02	0.23 ± 0.02	72.56 ± 0.96
R2	23.13 ± 0.59	11.24 ± 0.38	1.51 ± 0.09	0.43 ± 0.03	0.81 ± 0.04	0.34 ± 0.02	37.46 ±0.65
R3	10.51 ± 0.31	4.21 ± 0.18	2.96 ± 0.13	0.32 ± 0.02	2.83 ± 0.12	0.89 ± 0.05	21.72 ± 0.41
R4	0.16 ± 0.01	0.04 ± 0.00	0.93 ± 0.06	0.23 ± 0.02	8.39 ± 0.24	1.07 ± 0.06	10.82 ± 0.28
R5	0.31 ± 0.02	0.07 ± 0.00	0.67 ± 0.04	0.19 ± 0.01	3.16 ± 0.15	0.56 ± 0.03	4.91 ± 0.22
R6	0.09 ±0.00	0.03 ± 0.00	0.88 ± 0.05	0.17 ± 0.01	17.89 ± 0.52	1.36 ± 0.07	20.42 ± 0.37

R1-stem; R2-axis; R3-inflorescence; R4-flower without seeds; R5-inflorescence stalk; R6-corolla. The data were presented as mean ± standard deviations of three experiments.

**Table 5:** IC_50_ value of different parts from cultivated *C. deserticola* in a DPPH radical scavenging assay (*n* = 3).

Sample	IC_50_ (μg/mL)
R1	53.659 ± 1.04
R2	88.615 ± 1.61
R3	125.836 ± 2.53
R4	123.986 ± 2.33
R5	202.46 ± 2.98
R6	79.087 ± 1.37
Trolox	2.18 ± 0.03
l-Ascorbic acid	1.78 ± 0.01

**Table 6:** Identification of six PhGs by UPLC-ESI-Q/TOF-MS.

Peak No.	RT (min)	Identification	λmax (nm)	MS Data (*m*/*z*)
3	7.22	Echinacoside	224.0, 330.9	785, 623, 477, 461, 315, 179, 161, 135
4	8.88	Cistanoside A	222.2, 331.5	799, 637, 491, 475, 179, 161, 135
5	10.99	Acteoside	222.8, 330.2	623, 461, 315, 179,161,135
6	12.35	Isoacteoside	224.2, 327.2	623, 461, 315, 179,161,135
8	14.44	2′-Acetylacteoside	222.8, 330.9	665, 623, 503, 461, 315, 179, 161, 135
9	15.09	Tubuloside B	222.2, 327.8	665, 623, 503, 461, 315, 179, 161, 135

**Table 7:**
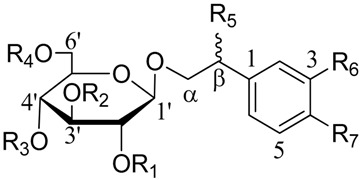
Chemical structure of six reference standards used in this study.

No.	Compounds	R1	R2	R3	R4	R5	R6	R7
1	acteoside	H	Rha	Cf	H	H	OH	OH
2	cistanoside A	H	Rha	Cf	Glc	H	OMe	OH
3	echinacoside	H	Rha	Cf	Glc	H	OH	OH
4	isoacteoside	H	Rha	H	Cf	H	OH	OH
5	2′-acetylacteoside	Ac	Rha	Cf	H	H	OH	OH
6	tubuloside B	Ac	Rha	H	Cf	H	OH	OH
